# Therapeutic hypothermia protects the ischemic penumbra via Xkr8

**DOI:** 10.1016/j.neurot.2026.e00948

**Published:** 2026-06-20

**Authors:** Zhanwei Zhu, Jian Chen, Jiaxin Hu, Jiachen He, Qi Liu, Jiaqi Guo, Yuncong Li, Jingbei Liu, Shichun Min, Shuaili Xu, Xi Chen, Xiaoduo He, Meimei Tao, Di Wu

**Affiliations:** a Department of Neurology and China-America Institute of Neuroscience, Xuanwu Hospital, Capital Medical University, Beijing 100053, China; b Department of Respiratory and Critical Care Medicine, Beijing Anzhen Hospital, Capital Medical University, China; c Beijing Institute of Brain Disorders, Capital Medical University, Beijing 100053, China

**Keywords:** Ischemic stroke, Penumbra, Therapeutic hypothermia, Apoptosis, Xkr8

## Abstract

Salvage of the ischemic penumbra is critical for improving outcomes after stroke, yet effective molecular targets remain elusive. Therapeutic hypothermia (TH) is a promising neuroprotective strategy, but its precise mechanisms, particularly concerning penumbral salvage, are not fully understood. Here, we demonstrate that TH significantly reduces infarct volume and limits the expansion of the ischemic penumbra in a murine model of transient middle cerebral artery occlusion. Mechanistically, TH exerted its protective effects primarily by suppressing apoptosis rather than other forms of regulated cell death. We identified phosphatidylserine (PS) exposure—a key “eat-me” signal for apoptosis—as a critical target of TH. TH selectively attenuated ischemia-induced PS externalization in neurons both in vivo and in vitro. Furthermore, we found that TH downregulated the expression of the scramblase Xkr8, a major regulator of PS exposure. Inhibition of Xkr8 mimicked the neuroprotective effects of TH, reducing PS exposure and improving neuronal survival, whereas overexpression of Xkr8 mitigated TH-mediated protection in the mouse model. Collectively, our findings reveal that TH protects the ischemic penumbra by inhibiting Xkr8-dependent PS exposure and subsequent apoptotic neuronal death, highlighting Xkr8 as a novel therapeutic target for ischemic stroke.

## Introduction

Acute ischemic stroke represents a significant threat to human health [1]. Vascular recanalization therapy has emerged as a crucial treatment modality for acute ischemic stroke [2]. However, outcomes remain suboptimal even when timely recanalization is achieved for stroke patients [3]. The ischemic penumbra region, characterized by a substantial number of potentially salvageable neurons, is the key to stroke treatment [4]. Consequently, preventing the transformation of the ischemic penumbra into new infarctions is a critical challenge in the management of ischemic stroke [5]. Previous studies have identified some protective targets for penumbra, such as NMDA antagonists and NXY-059, however, their clinical translational efficacy remains unsatisfactory [[6], [7], [8]]. Therefore, there is an urgent need to discover new targets and mechanisms for penumbra protection [[9], [10], [11]]. The key to protecting the ischemic penumbra is to prevent further damage and death of neurons in the penumbra after stroke [4].

Notably, therapeutic hypothermia (TH) is considered a pleiotropic neuroprotective intervention, capable of alleviating metabolic stress, excitotoxicity, inflammation, and apoptosis after ischemia. TH has shown great promise in both animal and clinical studies [12]. TH has been shown to effectively reduce the volume of cerebral infarction and improve the neurological outcomes of stroke patients [13]. However, no single existing target or mechanism fully explains the protective effects of TH. Thus, there might be some new mechanisms of TH pleiotropy. It is of great interest to explore whether TH reduces infarct size in patients by affecting the development of ischemic penumbra.

Phosphatidylserine (PS), a molecule typically localized on the inner leaflet of the cell membrane, is translocated to the outer leaflet under cellular stress conditions. PS is recognized as the most conserved “eat me” signal and believed to significantly influence apoptosis in cells. This externalized PS serves as an “eat me” signal, which will be recognized by macrophages, leading to subsequent phagocytosis of the stressed cell [14]. To date, three proteins have been identified as key regulators of PS exposure: TMEM16F, TMEM30A, and Xkr8 [15]. However, the mechanism of PS in stroke is still not well understood, especially after TH. Whether changes in the penumbra can be affected through PS-mediated apoptotic pathways is still unclear.

In this study, we investigated the underlying mechanisms of TH in protecting the penumbra and identified novel therapeutic targets for penumbra intervention.

## Materials and Methods

### Animal model

All animal experiments were performed at XuanWu Hospital, Capital Medical University and were approved by the XuanWu Hospital Institutional Animal Care and Use Committee. We strictly followed the National Institutes of Health Guide for the Care and Use of Laboratory Animals, and the experiments reported followed the ARRIVE guidelines. Male 8-10-week-old C57BL/6J mice purchased from SPF (Beijing) were housed in a specific pathogen-free facility with a 12-h light/dark cycle at XuanWu Hospital. Mice were randomly assigned to MCAO, MCAO + TH, or sham control groups using a lottery box. Mice were anesthetized with 1.5% isoflurane in a 30% O2/68.5% N_2_O mixture. MCAO was induced in the right hemisphere for 60min as described in previous publications. In brief, cerebral blood flow (CBF) was monitored with a laser-speckle blood flow imaging system (RWD Life Science) at four time points: baseline, after occlusion, before reperfusion, and after reperfusion. Mice that showed an approximately 70% drop in CBF were included in the infarct volume analysis. Reperfusion was achieved by withdrawing the filament 60 min after occlusion in mice. Surgeries and stroke outcome assessments were performed by investigators blinded to the experimental group assignments.

### Therapeutic hypothermia and temperature monitoring

Local Therapeutic hypothermia (TH) was induced immediately after ischemic onset. Hypothermic treatment was administered based on our previous protocols with some modifications [16]. In brief, an ice box was placed on the parietal and occipital parts of the mouse head. Body temperature was continuously monitored using a rectal probe and maintained at 36.5–37.5 °C with a regulated heating pad (Physitemp; TCAT-2LV controller). In a pilot study, the temperatures of the striatum in the infarct hemisphere were measured with a thermometer. One temperature probe (needle thermistor probes, Harvard Apparatus Inc.) was inserted at a depth of 3.0 mm at 0.86 mm anterior to the bregma and 2 mm lateral to the sagittal suture to assess striatal temperature. Another probe was inserted into the gap between the temporalis and the skull. The baseline temperature and the temperatures during hypothermia and rewarming were all recorded. To achieve the target temperature in the striatum (between 34 and 36 °C), we discovered that the temperature of the subtemporalis muscle had to be maintained between 33.1 and 35.1 °C. Thus, in subsequent experiments, only the subtemporalis temperature was monitored, without insertion of an invasive probe directly into the brain. The baseline temperature and the temperatures during hypothermia and rewarming were all recorded. Local TH was performed on mouse models of tMCAO immediately after the onset of 1 h occlusion and continued to 1 h after the reperfusion.

### Laser speckle imaging

Mice were anesthetized and focal ischemic was induced using the filament method. The scalp and the overlying membrane from the skull were gently incised down the midline and peeled to the side. A laser diode (780 nm) was used for illumination and laser speckle images were recorded with a CMOS camera (acA1300-200μm, Basler), 25 min after occlusion. For each animal, three sets of raw speckle images were acquired in <15 s (300 frames in each set; image width, 1280 pixels; image height, 1024 pixels; pixel size, 11.72 μm; exposure time, 5 ms). A speckle contrast image was calculated from each raw speckle image using a sliding grid of 5 × 5 pixels. A mean speckle contrast image was calculated for each set and used to calculate the decorrelation time and relative cerebral blood flow (rCBF) at each pixel. The blood flow (rCBF) at each location was standardized compared with that at baseline. The mean lateral-medial profile of the rCBF was computed for the 4.1–0.6-mm range from the midline, within a coronal band between lambda and bregma. We defined the ischemic penumbra as the cortical territory with rCBF values between 30 and 50% of baseline and estimated the width of the penumbra from the mean lateral-medial rCBF profile. These steps were repeated for each acquisition set. These thresholds were based on previously published operational definitions. The ischaemic threshold for infarction (that is, the core) is approximately 20–25 ml/100 g/min. The threshold for gene expression and protein synthesis inhibition (that is, upper limit for penumbra) is approximately 50–60 ml/100 g/min. Based on rodent CBF values of approximately 110–120 ml/100 g/min, this corresponded to a penumbral range of 30–50%. Speckle imaging data are expressed as relative flow values, CBF data from all pooled hemispheres were plotted as histograms. All analysis was done using a custom script written in Graphpad Prism 10.1.2. All analyses were randomized and operators were blinded.

### Western blotting

The mouse brain tissues were lysed with RIPA buffer, which includes protease inhibitor and phosphatase inhibitor. Using the bovine serum albumin (BSA) protein assay kit (Applygen Technologies Inc., Beijing, China) that protein concentration was determined. After separating the proteins (50 μg/lane) on a 10 % SDS-PAGE gel, they were electro-transferred to PVDF membranes (Millipore, Bedford, MA, USA). They were then blocked in Tris-buffered saline (pH 7.4) consisting of 5 % non-fat milk at room temperature for 2 h. The membranes were incubated with primary antibodies overnight at 4 °C, including anti-TMEM30A (1:1000, ab217330, Abcam), XKR8 Polyclonal Antibody (1:1000, PA5-98929, Thermofisher Scientific), Polyclonal Antibody to Anoctamin 6 (1:1000, PAF813Hu01, Cloud-Clone, Wuhan, China). Membranes then underwent three washes with suitable HRP-conjugated secondary antibodies (Zhongshan Biotechnology Co.) at room temperature for 2 h. Thereafter, a chemiluminescence detection kit (Zhongshan Biotechnology Co) was used to visualize the antigen-antibody complexes. The images were obtained from the density of all bands per group of target protein were averaged and used for statistical analysis. **Detailed information on all antibodies used in this study is provided in** Table S1**.**

### Immunofluorescence

For brain immunofluorescence staining analysis, mice were anesthetized and transcardially perfused with paraformaldehyde (PFA). The brains were dissected, postfixed in 4% PFA at 4 °C for 2 d, and then soaked in 30% sucrose solution at 4 °C for 4 d, sectioned into 20-μm-thick slices using the vibratome (Leica). The brain sections were blocked with 10% normal donkey serum and 1% Triton X-100 in phosphate-buffered saline (PBS, pH 7.4) for 1 h, and incubated with the anti-TMEM30A (1:200, ab217330, Abcam), XKR8 Polyclonal Antibody (1:200, PA5-98929, Thermofisher Scientific), Polyclonal Antibody to Anoctamin 6 (1:200, PAF813Hu01, Cloud-Clone, Wuhan, China) at 4 °C overnight. After washing with PBS, the samples were incubated with appropriate secondary antibodies conjugated to Alexa Fluor 488 or Alexa Fluor 594 (550043,ZENBIO, Chengdu,China) at room temperature for 1 h. The sections were counterstained with DAPI for 30min, followed by washing in PBS. Images were acquired by confocal microscopy. **Detailed information on antibodies used for immunofluorescence staining is provided in** Table S1**.**

### Primary cortical neuron culture and oxygen-sugar deprivation (OGD)

Primary cortical neurons were prepared from embryonic day 18 mice as previously described. Briefly, cerebral cortices were isolated under sterile conditions, meninges were removed, and tissues were digested with trypsin at 37 °C followed by gentle mechanical dissociation. Cells were seeded onto poly-d-lysine-coated dishes or coverslips and cultured in DMEM containing 10% fetal bovine serum and 10% horse serum for 4 h. The medium was then replaced with Neurobasal medium supplemented with 5% B27 and 0.5 mM GlutaMAX, and neurons were maintained at 37 °C in 5% CO_2_. Half of the culture medium was replaced periodically, and neurons were cultured for 5 days before experiments [17].

For oxygen-glucose deprivation (OGD), neurons were transferred to glucose-free DMEM and incubated in a hypoxic chamber containing 1% O_2_, 94% N_2_, and 5% CO_2_ for 120 min, followed by recovery under normoxic conditions in standard Neurobasal medium.

### 2,3,5-Triphenyl tetrazolium chloride (TTC) staining

The mouse brain was cut into coronal sections (1 mm), and then stained with 2% TTC (T8170, Solarbio, Beijing, China) for 10 min at 37 °C. ImageJ software (US National Institutes of Health, Bethesda, USA) was used to measure the volume of non-infarcted tissues (stained red), calculated as: Infarct volume (%) = (the volume of control hemisphere − the non-infarcted volume of tMCAO hemisphere)/(2 × the volume of control hemisphere).

### Quantitative polymerase chain reaction (PCR)

Total RNA was extracted from fresh brains using TRIzol reagent (R411-01, Vazyme) according to the manufacturer's guidelines. Then, cDNA synthesis was conducted using HiScript II Q RT SuperMix (R223-01, Vazyme). Quantitative PCR was performed using ChamQ SYBR qPCR Master Mix (Q311-02, Vazyme) and an ABI QuantStudio3 Real-Time PCR Detection System (Thermo Fisher Scientific, Waltham, USA). The amplification cycle of target gene was normalized to that of the housekeeping gene Actin and relative gene expression was calculated using the 2−ΔΔCt method.

### Magnetic resonance imaging

Mice were imaged on a horizontal-bore 7.0 T small-animal MRI (volume transmit/surface receive coil) under isoflurane anesthesia (induction 3–4%, maintenance 1–1.5% in O_2_). Body temperature was maintained at 37.0 ± 0.5 °C with a circulating-water heating pad and respiration and temperature were continuously monitored and gated. Multislice T2-weighted rapid acquisition with relaxation enhancement (RARE) images were acquired (TR/TE = 3000/40 ms, RARE factor = 8, FOV = 16 × 16 mm, matrix = 128 × 128, in-plane resolution = 125 × 125 μm, slice thickness = 0.5 mm, 20–24 contiguous slices). Total scan time per animal was 10 min. Images were inspected for motion and susceptibility artifacts, datasets with major artifacts were excluded. Infarct volumes were delineated on T2-weighted images by intensity thresholding and manual correction, and corrected for edema using the contralateral-hemisphere method. ADC maps were used to confirm cytotoxic injury. All image processing and volumetric measurements were performed in ImageJ or ITK-SNAP by an operator blinded to treatment group, results are reported as mean ± SEM.

### Flow cytometry

Primary cortical neurons were harvested by gentle trituration, washed twice in ice-cold PBS, and resuspended at 0.5–1.0 × 10ˆ6 cells/mL in 1 × Annexin V binding buffer (10 mM HEPES, 140 mM NaCl, 2.5 mM CaCl_2_, pH 7.4). For apoptosis/viability assessment, cells (100 μL) were incubated with Annexin V-FITC (5 μL per 100 μL) for 15 min at room temperature in the dark. Immediately prior to acquisition, 7AAD was added at 1 μg/ml and samples were kept on ice and protected from light. Samples were analyzed within 1 h on a flow cytometer equipped with a 488 nm laser, FITC fluorescence was detected using a 530/30 nm bandpass filter and 7AAD with a >650 nm detector. Forward/side scatter gating excluded debris and doublets, live (Annexin V-/7AAD-), early apoptotic (Annexin V+/7AAD-), late apoptotic/necrotic (Annexin V+/7AAD+), and necrotic (Annexin V-/7AAD+) populations were quantified from ≥10,000 events per sample. Data were compensated and analyzed using FlowJo and are reported as mean ± SEM from at least three independent cultures [18].

### Bederson neurological score

Neurological deficits were assessed 24 h after MCAO using the Bederson scale. Mice were gently lifted by the tail and observed for forelimb posture, body torsion, and resistance to lateral push. Scoring was performed as follows: 0, no deficit; 1, forelimb flexion; 2, decreased resistance to lateral push without circling; 3, spontaneous circling; 4, no spontaneous movement. Each animal was evaluated for ≥10 s in a quiet environment by an investigator blinded to treatment. Three consecutive assessments were averaged to obtain the final score [19].

### Balance Beam Test

Motor coordination was examined on a 1-cm-wide, 80-cm-long elevated beam (50 cm above the platform). Performance was assessed by counting foot slips and total traversal time. A maximum cut-off of 60 s was applied. Three trials per mouse were averaged. All analyses were conducted by an investigator blinded to group allocation.

### Corner test

Sensorimotor asymmetry was evaluated using the corner test. Mice were placed in a 30° angled corner lined with two vertical boards. When entering the corner, mice turned either left or right; a preferential turn toward the non-impaired side indicates neurological deficit. Each mouse performed 3 trials, with turning direction recorded for each trial. The percentage of turns toward the ipsilateral side was calculated. Animals were tested in a quiet room and scored by blinded a investigator [20].

### Foot-fault test

The foot-fault test, also named grid-walking task, was used to assess a rodent sensorimotor function impairment after ischemic stroke. The grid-walking apparatus was manufactured with minor modification, a 24 rid apparatus measuring 40∗20∗50 cm (length/width/height) with a mesh size of 12 mm. Each mouse was placed individually atop the wire grid and allowed to walk freely for 5 min, 24 h/3 d/7d after MCAO surgery a foot fault was recorded. A step was considered foot-fault if the limb dropped into the grid hole or if animals rested with the grid at the level of wrist. The total number of steps and foots faults was recorded by investigators blinded to the experiment. Each mouse completed two trials. Results were averaged by a blinded assessor. The ratio of foot faults was calculated by the following formula: foot faults/(foot fault + non-foot fault steps) × 100% [21].

### Rotarod test

Motor coordination and balance were tested using an accelerating rotarod (Ugo Basile). Mice were pre-trained for 7 days before MCAO. For testing, the rod accelerated from 4 to 40 rpm over 5 min. Latency to fall was recorded, with a maximum cutoff of 300 s. Each mouse performed three trials with ≥15 min rest between trials; the mean latency was used for analysis. All testing and scoring were performed by an investigator blinded to treatment [21].

### Statistical analysis

Statistical analyses were performed as previously described [[22], [23], [24]]. GraphPad Prism version 10.1.2 was used for statistical analysis. The experimental data are shown as the mean ± standard error of the mean (SEM). For statistical comparisons, an independent two-sample Student's t-test (between two groups) and one-way ANOVA (between multiple groups) with Tukey's test were employed for normally distributed data with equal variances. For behavioral assessments performed across multiple time points (Balance Beam Test, Corner Test, Foot-Fault Test, and Rotarod Test), data were analyzed using two-way repeated-measures ANOVA with treatment as the between-subject factor and time as the repeated-measures factor, followed by Tukey's multiple-comparisons test. The p value < 0.05 was considered to indicate statistical significance.

### Adeno-associated virus transduction in vivo

Adeno-associated virus (AAV) delivery was performed as previously described [25]. Briefly, mice were anesthetized and placed in a stereotaxic apparatus for intracerebroventricular (ICV) injection. AAV vectors were injected into the lateral ventricle at coordinates relative to bregma (approximately 0.3 mm posterior, 1.0 mm lateral, and 2.5–3.0 mm depth) using a microsyringe. A total volume of 3–5 μL AAV (approximately 1 × 10ˆ13 GC/ml) was slowly injected to minimize tissue damage and reflux. After injection, the needle was left in place for 5–10 min before withdrawal. Mice were then sutured, allowed to recover, and maintained for sufficient viral expression before subsequent experiments.

## Results

### Therapeutic hypothermia protects the ischemic penumbra mainly by inhibiting apoptosis

**Fig. 1:**
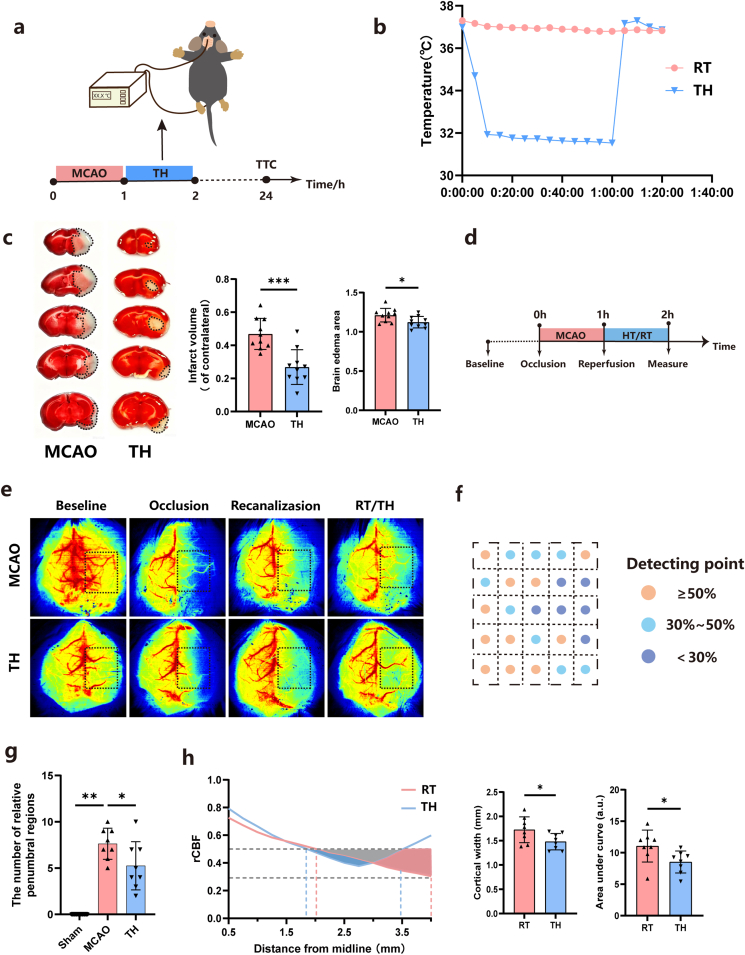
Therapeutic hypothermia (TH) protected the ischemic penumbra. (a) Construction of a local low temperature detection system. Top: An electrode is inserted into the skull to measure brain temperature, and an electrode is inserted into the anorectum to measure core temperature. Bottom: Time flow chart. (b) TH intervention or RT intervention lasted for 1 h, and the brain temperature of mice was measured every 5 min (n = 5). (c) After 24 h of reperfusion, the staining results of TTC brain slices treated with TH or RT, as well as the statistical results of infarct size and brain edema area were analyzed (n = 10). (d) Flow chart of laser speckle imaging experiments. (e) The results of the TH and RT treatments of the laser speckle imaging experiment, the black dashed rectangle represents the brain regions used for statistics. (f) A total of 25 fixed points were detected, and the relative cerebral blood flow at each point was calculated to estimate the area in the normal/penumbra/infarct core. (g) Statistics of the number of brain regions in the penumbra after RT and TH treatment (n = 8). (h) Left: Statistical plot of blood flow gradients from the brain midline to the brain periphery after RT treatment and TH treatment. Middle: Comparison of widths of brain regions in the penumbra region calculated from the left panel. Right: Comparison of brain areas in penumbra regions calculated from the left image (n = 8). RT: Room temperature. TH: Therapeutic hypothermia. ∗*P* < 0.05, ∗∗*P* < 0.01, ∗∗∗*P* < 0.001.

Therapeutic hypothermia (TH) has been widely acknowledged as an effective neuroprotective strategy following stroke [16,26,27]. However, fewer studies have focused on TH-specific protective mechanisms. Here, male C57BL/6 mice underwent transient middle cerebral artery occlusion (MCAO) for 60 min, after which they were either subjected to TH treatment or maintained at room temperature (RT). The skull surface and rectal temperatures were continuously monitored using a thermal detection system as previously reported [16] (Fig. 1a). Cortical temperature was maintained at approximately 31.8 °C for 60 min, while rectal temperature remained around 37.8 °C (Fig. 1b). Their brains were removed 24 h after the onset of occlusion. TH reduced cerebral infarction volume and brain edema when compared with RT controls (Fig. 1c).

Ischemic penumbra, as a salvageable brain tissue after stroke, is the main target for the treatment of ischemic stroke [28,29]. We evaluated the development of ischemic penumbra after TH treatment. Laser speckle imaging was used to monitor cerebral blood flow and calculate the ischemic penumbra (defined as 30%–50% of baseline cerebral blood flow perfusion) [30,31]. Baseline cerebral blood flow (CBF) in each region was measured prior to MCAO for subsequent relative CBF calculations. Mice were subjected to MCAO for 1 h, followed by reperfusion treatment, and 1 h TH or equivalent RT (Fig. 1d–e). The relative CBF was measured and calculated in the same regions of the mouse brain at different times. The number of areas in the relative penumbra was used to estimate the area of ischemic penumbra and determine the change of penumbra (Fig. 1f). Compared with the Sham group, the number of brain regions in the penumbra region was significantly increased in the MCAO + RT group. TH significantly reduced the development of penumbra after stroke onset (Fig. 1g). We calculated the critical perfusion area relative to the central axis to estimate the ischemic penumbra according to a previously established method [32]. The perfusion gradient was observed in the ipsilateral cortex, from an approximately normal level in the midline to a significantly reduced level within the infarct core (Fig. 1h). This gradient was markedly attenuated in TH-treated mice compared with RT controls (Fig. 1h). The ischemic penumbra was significantly smaller in TH-treated animals than in RT controls (Fig. 1h). These findings suggest that TH may inhibit the expansion of the ischemic penumbra.

**Fig. 2:**
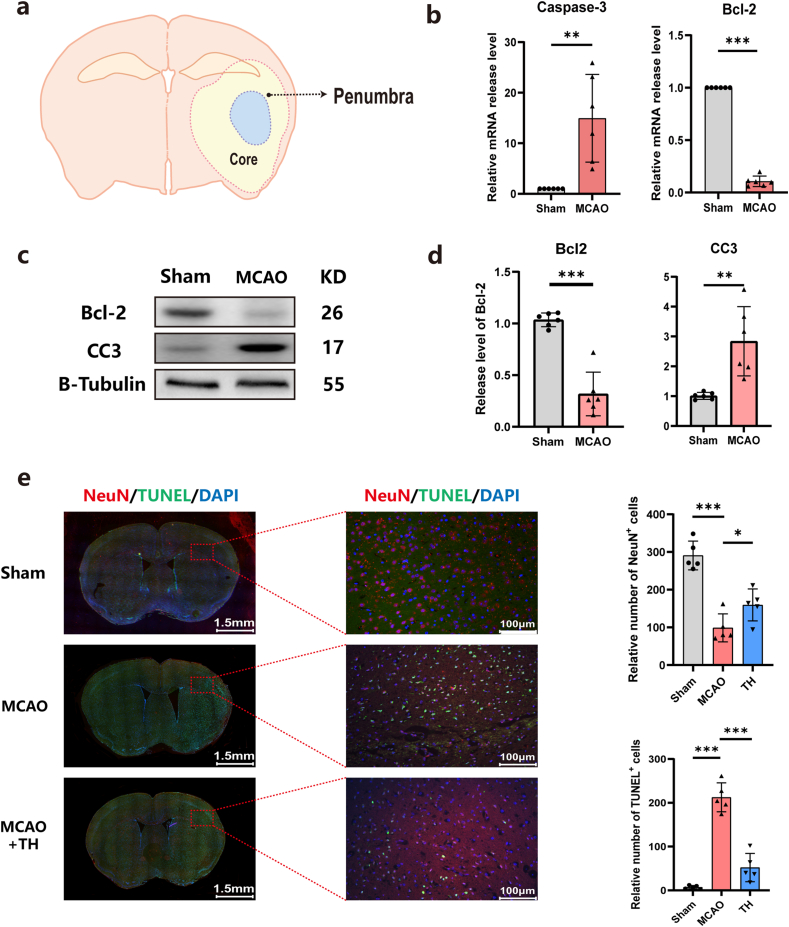
The form of cell death in the ischemic penumbra region is mainly apoptosis. (a) Examined brain regions used for experimental validation. (b) Statistical results of relative mRNA levels of Caspase-3 and Bcl2 in the ischemic penumbra region at 24 h of reperfusion (n = 6). (c–d) Statistical results of relative protein levels of Cleaved Caspase-3 (CC3) and Bcl2 in the ischemic penumbra region at 24 h of reperfusion (n = 6). (e) Results of immunofluorescence analysis of NeuN and TUNEL (n = 5). ∗*P* < 0.05, ∗∗*P* < 0.01, ∗∗∗*P* < 0.001.

Recent studies have identified several novel forms of regulated cell death, including pyroptosis [33,34], necroptosis [35], and ferroptosis [36] besides apoptosis. We also used established molecular markers to determine the mode of cell death that may play a dominant role in the ischemic penumbra region of mice after MCAO. Apoptosis was evaluated with Caspase-3 and Bcl-2 [37], pyroptosis with Caspase-1 and GSDMD [38], necroptosis with RIPK3 and MLKL [39], and ferroptosis with ACSL4 and GPX4 [40,41]. Brain tissue from the ischemic penumbra region was harvested after 24 h reperfusion (Fig. 2a). MCAO upregulated mRNA expression of the pro-apoptotic gene Caspase-3, and downregulated the anti-apoptotic gene Bcl-2, when compared to control (Fig. 2b). In contrast, no significant changes were observed in Caspase-1 mRNA levels during pyroptosis assessment, although GSDMD expression was markedly reduced. For necroptosis, neither RIPK3 nor MLKL showed significant differences in mRNA levels relative to the sham group. Similarly, in the case of ferroptosis, no significant alterations were detected in the expression of ACSL4 or GPX4 (Fig. S1 a-c). For further validation, these key factors were tested at the protein level. After MCAO, the protein level of Cleaved Caspase 3 was significantly increased and the protein level of Bcl-2 was significantly decreased in the ischemic penumbra region (Fig. 2c–d), but no statistically significant differences in the protein levels of caspase-1, GSDMD, RIPK3, MLKL, ACSL4 and GPX4 were observed (Fig. S1 d-e). These data suggest that the dominant form of death in the 24 h ischemic penumbra of MCAO may be apoptosis.

Double immunofluorescence staining for NeuN and TUNEL showed that ischemia led to an increased number of TUNEL^+^ cells and decreased number of NeuN^+^ cells in the ischemic penumbra region when compared with the control group. However, TH significantly reduced the number of TUNEL^+^ cells and improved neuronal survival (Fig. 2e).

Taken together, these results suggest that TH may inhibit penumbra development and improve neuronal survival by reducing apoptosis in the ischemic penumbra.

### PS as a critical “eat-me” signal mediating the protective effects of therapeutic hypothermia

**Fig. 3:**
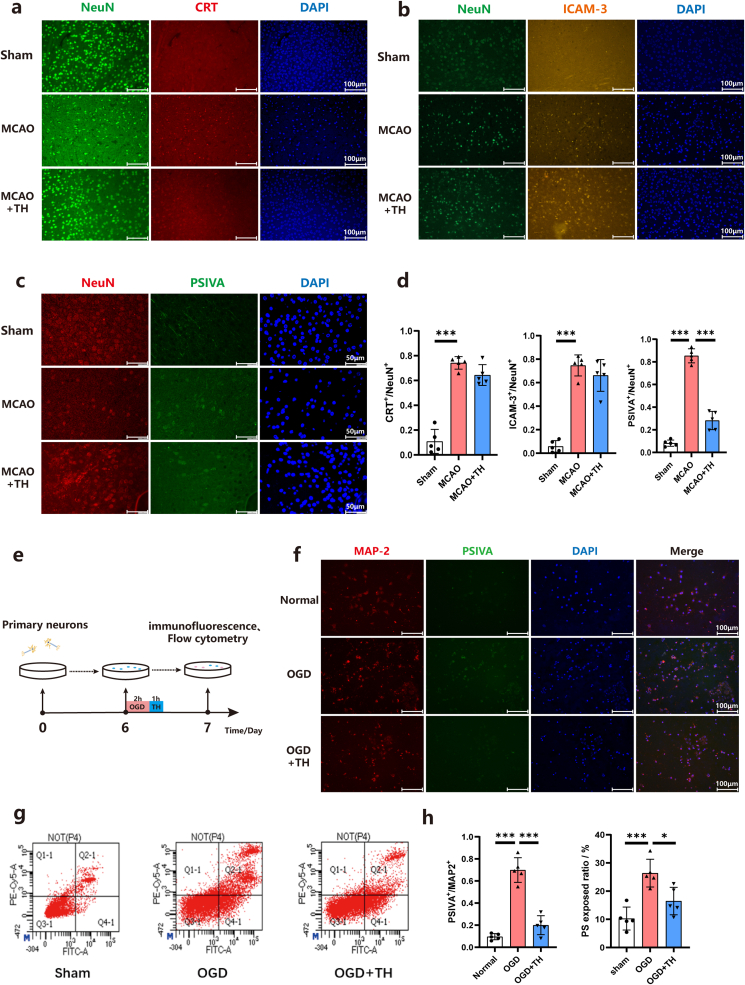
Phosphatidylserine (PS) may be a key target for the protective effect of TH, TH significantly inhibited PS exposure in OGD neurons in vitro. (a–d) The results of CRT, ICAM-3 and PS were analyzed by immunofluorescence in the ischemic penumbra (n = 5). (e) Temporal flowchart of primary neuron extraction and culture. (f) After 24 h of OGD/R in primary neurons, results of immunofluorescence analysis of PSIVA. (g) After 24 h of OGD/R in primary neurons, results of flow cytometry analysis of PSIVA. (h) Statistical results of immunofluorescence (left) and flow cytometry (right) (n = 5). ∗*P* < 0.05, ∗∗*P* < 0.01, ∗∗∗*P* < 0.001.

The “eat me” signal is considered an important component for the apoptotic signaling pathways [15]. Previous studies have proposed intercellular adhesion molecule-3 (ICAM-3), calreticulin (CRT), and PS as three key “eat me” signals [15]. However, the involvement of “eat me” signal in the neuroprotection of TH remains less explored. To evaluate this hypothesis, we assessed the expression of these three signals, ICAM-3, CRT, and PS in penumbral regions. The immunofluorescence staining of brain sections indicated that CRT, ICAM-3, and PS in the penumbra were significantly increased in MCAO group compared with those in Sham group (Fig. 3a–c). However, only the TH treatment reduced PS exposure, but not CRT and ICAM-3 expression (Fig. 3d).

To minimize potential confounding effects due to the complex in vivo analysis, we also cultured primary neurons. The mature primary neurons were subjected to oxygen glucose deprivation (OGD) for 1 h, and additional TH was also administered to the treatment group (Fig. 3e). Immunofluorescence staining demonstrated that TH treatment markedly inhibited OGD-induced PS exposure in primary neurons (Fig. 3f–h). Furthermore, we performed Flow cytometry analysis to assess PS exposure. TH treatment reduced Annexin A5 positive (PS exposure) neurons when compared with controls (Fig. 3g–h). Collectively, our data indicate that TH treatment may exert neuroprotective effects by suppressing ischemia-induced neuronal PS exposure and reducing apoptotic cell death.

### Xkr8 may be a key molecule affecting PS exposure during TH

**Fig. 4:**
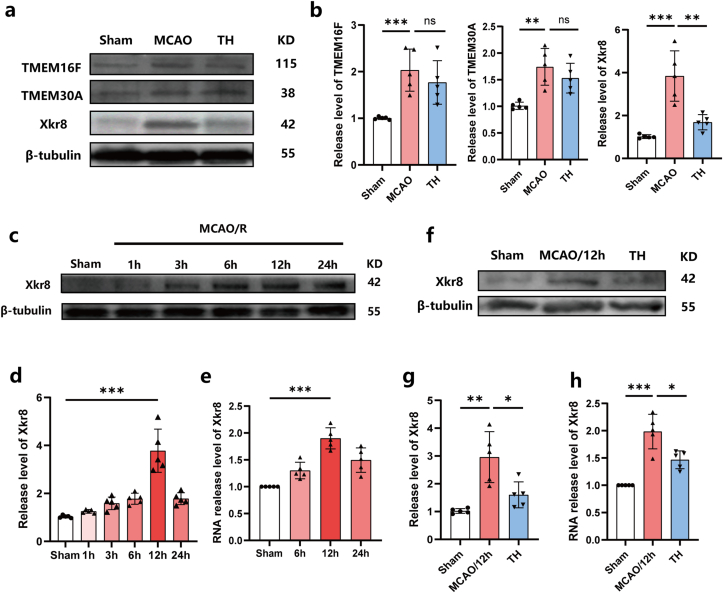
Xkr8 is a key factor in TH affecting PS exposure in the ischemic penumbra region. (a–b) Western Blot results of TMEM16F, TMEM30A and Xkr8 and statistical plots (n = 5). (c–d) Results of Western Blot analysis of Xkr8 protein expression at different time points after MCAO/R (c) and statistical plots (d) (n = 5). (e) Results of qPCR for Xkr8 mRNA levels at different time points after MCAO/R (n = 5). (f–g) TH significantly inhibited the protein expression of Xkr8 after 12 h of MCAO/R. (n = 5). (h) TH significantly inhibited the mRNA expression of Xkr8 after 12 h of MCAO/R (n = 5). ∗*P* < 0.05, ∗∗*P* < 0.01, ∗∗∗*P* < 0.001.

The externalization of PS is primarily regulated by flippase and scramblase [42]. Flippase, predominantly represented by P4-ATPases, mediates the translocation of PS to the inner leaflet of the cell membrane, with TMEM30A serving as a key auxiliary subunit. Scramblase, mainly including TMEM16F and Xkr8, facilitates the non-specific movement of PS between the inner and outer leaflets of the membrane [15]. To investigate the impact of TH on PS exposure and its potential neuroprotective mechanisms, Western blot analysis was performed to evaluate the expression levels of these key regulatory proteins. The results showed that cerebral ischemia upregulated the protein levels of TMEM16F, TMEM30A and Xkr8, TH treatment did not significantly affect the protein expression of TMEM30A or TMEM16F in the ischemic penumbra of MCAO mice (Fig. 4a–b). In contrast, TH treatment markedly reduced the protein expression of Xkr8 (Fig. 4a–b).

To further characterize the temporal dynamics of Xkr8 expression following TH in MCAO mice, time-course analyses were conducted. Xkr8 protein levels were found to peak at 12 h after reperfusion (Fig. 4c–d). This observation was corroborated by qPCR, in which Xkr8 mRNA levels in the ischemic penumbra also peaked at 12 h post-reperfusion (Fig. 4e). To assess whether TH modulates Xkr8 expression at this critical time point, we examined Xkr8 levels in MCAO mice treated with TH at 12 h after reperfusion. TH treatment significantly suppressed both Xkr8 protein and mRNA expression in the ischemic penumbra at this stage in the mouse model (Fig. 4f–h). These findings indicate that TH treatment may confer neuroprotection, at least in part, through the downregulation of Xkr8 expression in ischemic brain tissue.

### Inhibition of Xkr8 expression can reduce PS exposure and protect neurons

**Fig. 5:**
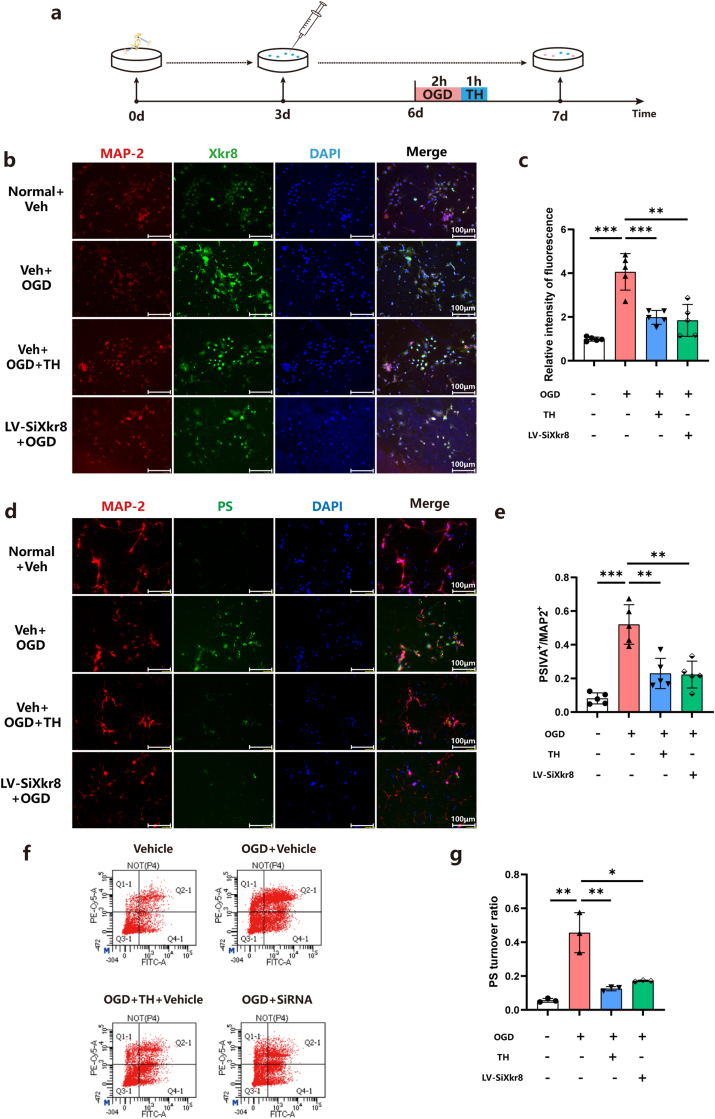
Inhibition of Xkr8 expression reduced PS eversion and improved neuronal survival. (a) Flowchart of primary neuron extraction, culture, and lentivirus transfection. (b–c) Plot (b) and statistical plot (c) of immunofluorescence staining results of primary neurons of Xkr8 (n = 5). (d–e) Plot (d) and statistical plot (e) of immunofluorescence staining results of primary neurons in PS (n = 5). (f–g) Results of PS exposure in primary neurons by flow cytometry (f) and statistical plots (g) (n = 3). ∗*P* < 0.05, ∗∗*P* < 0.01, ∗∗∗*P* < 0.001.

To assess the impact of Xkr8 on neuronal survival after MCAO, we transfected primary neurons with Xkr8-specific lentiviral vectors (LV) to suppress its expression. We first constructed three lentiviruses and identified the most effective one for Xkr8 knockdown using western blotting. LV-SiXkr8 (SiRNA3) showed the highest efficacy (Fig. S2a). We isolated primary neurons from embryonic mice. After 3 days in culture, neurons were transfected with LV-SiXkr8. On day 6, cultures underwent 2 h of oxygen-glucose deprivation (OGD), followed by 1 h of TH treatment or incubation at 37 °C. All assays were performed on day 7 (Fig. 5a). The results of immunofluorescence experiments showed that the LV-SiXkr8 group significantly reduced Xkr8 expression and enhanced neuronal survival relative to the control group (Fig. 5b–c). Additionally, the TH-Veh group showed similar Xkr8 inhibition effects and improvements in neuronal survival as the LV-SiXkr8 group. (Fig. 5b–c). To elucidate whether the neuroprotective effect of Xkr8 inhibition is mediated through the suppression of PS exposure, further experiments were conducted. The results showed that LV-SiXkr8 group significantly inhibited the increased PS exposure induced by OGD and improved the survival rate of neurons (Fig. 5d–e). Similarly, the TH-Veh group showed similar suppression of PS exposure and increased neuronal survival as the LV-SiXkr8 group (Fig. 5d–e). To further validate our findings, we performed flow cytometry experiments. The results showed that the exposure rate of PS was significantly higher in the OGD-Veh group (Fig. 5f–g). However, the OGD-induced increase in PS exposure was significantly suppressed in the LV-SiXkr8 group (Fig. 5f–g). The TH-Veh group exhibited a similar suppression of PS exposure as the LV-SiXkr8 group (Fig. 5f–g). These findings suggest that Xkr8 may intervene in the survival state of neurons by interfering with PS exposure.

### Overexpression of Xkr8 in the brain abrogated the protective effect of TH on neurons in the ischemic penumbra

**Fig. 6:**
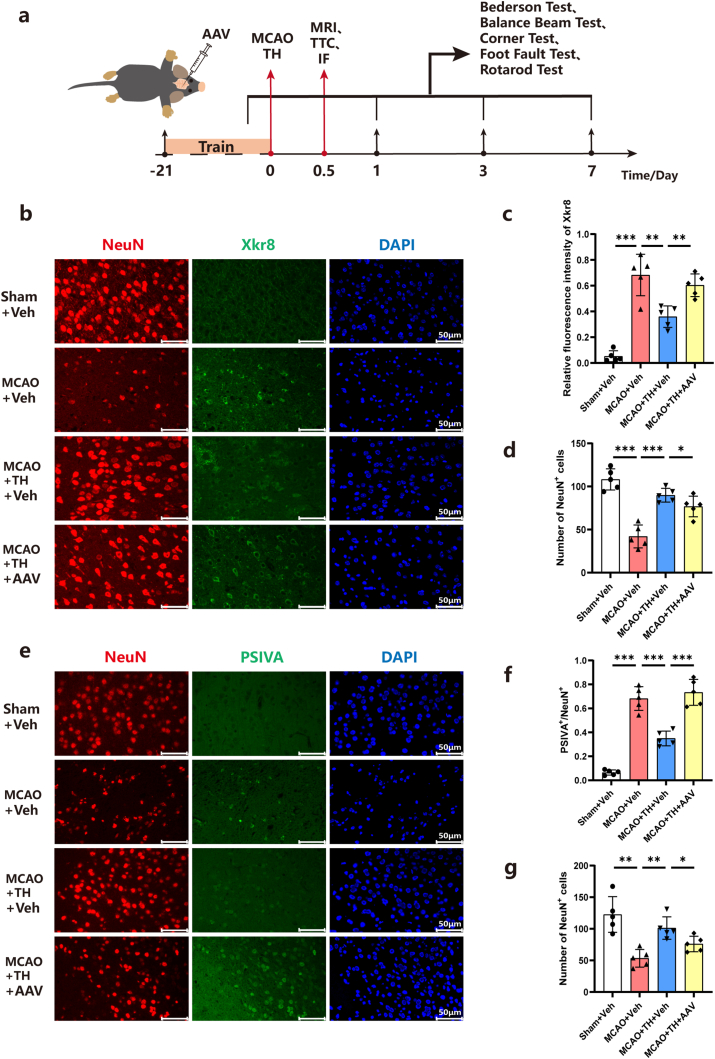
Xkr8 overexpression impaired TH inhibition of PS eversion and attenuated the improvement in neuronal survival. (a) Flow chart of mouse pre-surgical training, adeno-associated virus transfection, MCAO/TH and subsequent experimental detection. (b–d) Immunofluorescence results of Xkr8 and Neun co-staining, Xkr8 statistics and NeuN^+^ number statistics (n = 5). (e–g) Immunofluorescence results of PSIVA and NeuN^+^ co-staining, PSIVA statistics and NeuN^+^ number statistics (n = 5). ∗*P* < 0.05, ∗∗*P* < 0.01, ∗∗∗*P* < 0.001.

To investigate the functional role of Xkr8 in vivo, we employed adeno-associated virus (AAV) vectors to overexpress Xkr8 in neurons. First, to exclude potential off-target effects of stereotactic AAV injection, T2-weighted magnetic resonance imaging (MRI) was performed, which showed no structural abnormalities or unintended tissue damage in the targeted brain regions (Fig. S2b). Successful Xkr8 overexpression was confirmed by Western blot (Fig. S2c). On day 0, transient MCAO was induced for 1 h, after which mice were subjected to either TH or RT conditions for 1 h. A subset of animals was sacrificed at 12 h post-reperfusion, and brain tissues were collected for immunofluorescence staining, TTC staining, and MRI analysis focusing on the ischemic penumbra. Neurological deficits and motor function were evaluated using the Bederson scoring system, along with a behavioral test battery—including the balance beam, corner, foot-fault, and rotarod tests—at 1, 3, and 7 days after MCAO (Fig. 6a). Immunofluorescence results showed that TH + Veh group significantly reduced the expression of Xkr8 in mouse neurons after MCAO and improved the survival rate of neurons (Fig. 6b–d). However, this neuroprotective effect of TH was impaired when Xkr8 was overexpressed (Fig. 6b–d). Similarly, TH + Veh significantly reduced neuronal PS exposure after MCAO, but this phenomenon was also reversed when Xkr8 was overexpressed (Fig. 6e–g). These results suggest that overexpression of Xkr8 alleviates the neuroprotective effects of TH.

**Fig. 7:**
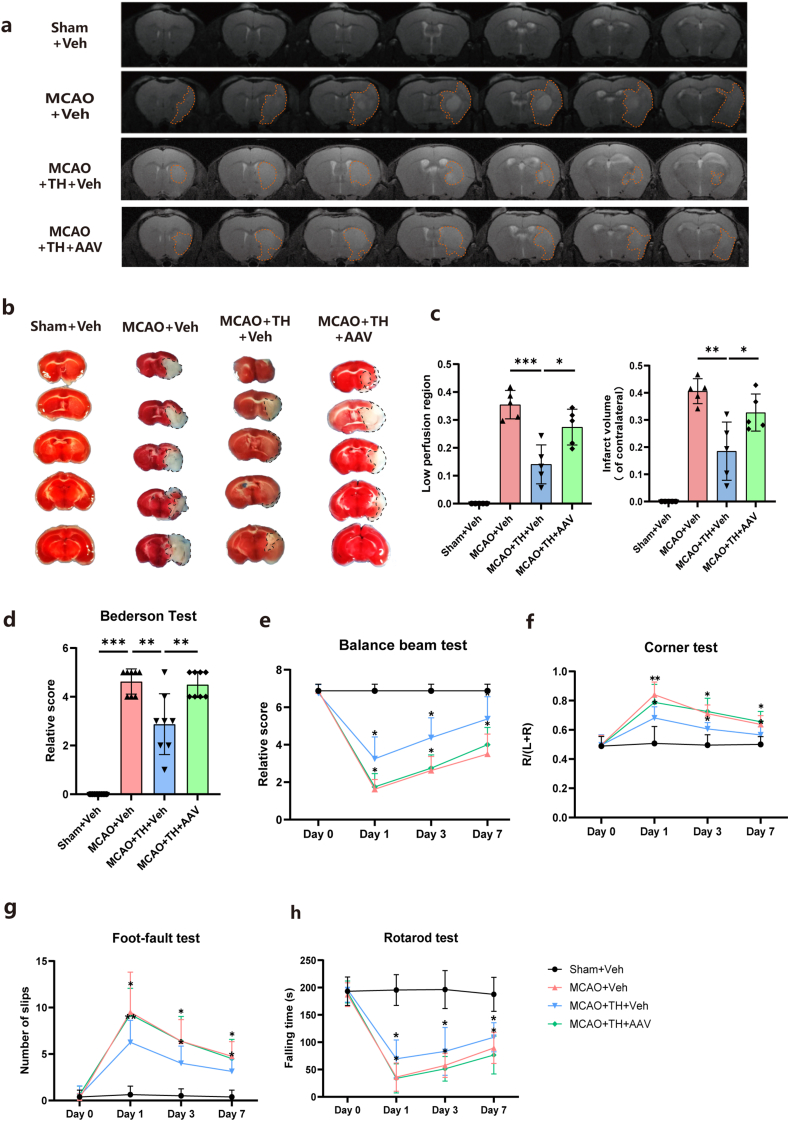
Overexpression of Xkr8 attenuated the improvement of cerebral protection by TH in MCAO mice. (a) The results of NMR examination in each experimental group. (b) TTC staining results for each experimental group. (c) Statistical plots of NMR and TTC results (n = 5). (d–h) Results of Bederson Test (d), Balance Beam Test (e), Corner Test (f), Foot-Fault Test (g) and Rotarod Test (h) in each experimental group. Data are presented as mean ± SEM (n = 8). ∗/**∗***P* < 0.05, ∗∗/**∗∗***P* < 0.01, ∗∗∗/**∗∗∗***P* < 0.001.

To definitively assess the impact of Xkr8 on functional outcomes after MCAO, we evaluated cerebral infarct volume and motor performance across experimental groups. Magnetic resonance imaging showed that TH + Veh group significantly reduced the area of cerebral infarction caused by MCAO (Fig. 7a,c). However, this reduction in infarct size was inhibited when Xkr8 was overexpressed (Fig. 7a,c). Similar results were observed in TTC staining of mouse brains. TH + AAV-Xkr8 attenuated TH-induced cerebral infarct volume reduction (Fig. 7b–c). Meanwhile, TH significantly improved the Bederson test score and motor performance in the Balance Beam Test, Corner Test, Foot-Fault Test, and Rotarod Test after MCAO (Fig. 7d–h). Across the observation period, these beneficial effects of TH were markedly attenuated by Xkr8 overexpression (Fig. 7d–h).

In conclusion, our experiments suggest that TH may inhibit PS everting in the “eat me” apoptotic signal by inhibiting Xkr8 expression in the ischemic penumbra region of mice, which in turn inhibits the development of ischemic penumbra, protects neuronal survival, and improves motor behavioral function in mice after MCAO.

## Discussion

Protection of the ischemic penumbra is a critical determinant of functional outcome after ischemic stroke. In the present study, our findings suggest that TH may limit ischemic penumbra expansion, reduce infarct volume, and improve neurological outcomes. Mechanistically, TH may preferentially attenuate neuronal apoptosis in the penumbra among multiple regulated cell death pathways. Importantly, TH may suppress ischemia-induced PS externalization in neurons, accompanied by downregulation of the scramblase Xkr8. Inhibition of Xkr8 produced effects similar to those observed following TH, whereas neuronal overexpression of Xkr8 partially attenuated hypothermia-associated neuroprotection. Collectively, these findings suggest that Xkr8-dependent PS exposure may represent an important molecular event linking TH to the suppression of neuronal apoptosis in the ischemic penumbra.

PS externalization is a conserved “eat me” signal that marks stressed or apoptotic cells for phagocytic clearance. Xkr8 has been identified as a principal caspase-activated scramblase responsible for irreversible PS exposure during apoptosis [43]. In parallel, emerging studies have shown that other scramblases, such as TMEM16F, can mediate reversible PS exposure in metabolically compromised yet potentially viable neurons, thereby promoting microglia-driven phagoptosis and progressive penumbral loss [44]. Within this framework, our study suggests a previously underappreciated mechanism by which TH may suppress Xkr8-mediated PS exposure in the ischemic penumbra. By integrating in vivo and in vitro approaches, we demonstrate that TH reduces PS externalization, downregulates Xkr8 at both the transcriptional and protein levels, and produces neuroprotection comparable to direct Xkr8 inhibition. In contrast, Xkr8 overexpression completely negates this protection. These results suggest that the scramblase Xkr8 may act as an important regulator of neuronal fate in the penumbra and may represent a potential target for neuroprotective intervention.

Therapeutic hypothermia is widely recognized as a pleiotropic intervention capable of influencing multiple pathological processes after ischemic stroke, including inflammation, mitochondrial dysfunction, oxidative stress, excitotoxicity, blood-brain barrier disruption, and metabolic imbalance [45]. The interactions among apoptotic signaling, inflammatory responses, and phagocytic clearance mechanisms may further complicate interpretation of direct mechanistic relationships [46,47]. In this study, the knockdown and overexpression experiments support the involvement of Xkr8 in TH-associated neuroprotection, the current evidence remains insufficient to establish a definitive causal relationship between TH-mediated protection and Xkr8-dependent PS externalization. Therefore, the neuroprotective effects of TH observed in the present study may not depend exclusively on modulation of the Xkr8-PS axis. Instead, Xkr8-dependent PS exposure may represent one component within a broader protective network associated with TH treatment.

Local and systemic hypothermia exhibit differences in both physiological effects and translational applicability [48]. Systemic hypothermia can provide broad metabolic suppression and neuroprotection after ischemic stroke; however, its clinical application is often limited by systemic complications, including shivering, cardiovascular instability, coagulation dysfunction, and increased infection risk [49]. In contrast, local hypothermia enables more targeted cooling of ischemic brain tissue while minimizing systemic adverse effects and reducing the time required to achieve therapeutic brain temperature, thereby showing greater translational potential for acute ischemic stroke treatment [50,51]. In this study, a local hypothermia strategy was adopted in the MCAO mouse model to preferentially reduce cerebral temperature while limiting whole-body physiological disturbances. This approach may facilitate a more direct evaluation of the neuroprotective effects of hypothermia on ischemic brain injury.

Furthermore, several limitations of our work should be acknowledged. First, our assessment of the size and volume of the ischemic penumbra was not entirely accurate. Due to technical limitations, our assessment of penumbra relied on a “two-dimensional” plane formed by relative cerebral blood flow, which prevented us from obtaining data on the fully true penumbra volume. Second, PS exposure is a dynamic transient event [52,53], and our sampling points may not capture early reversible exposure, thereby underestimating the full extent of PS dynamics in the penumbra. Third, although PS exposure is strongly associated with phagocytic clearance [54], we did not directly demonstrate microglial engulfment of PS-positive neurons, thus the loss of neurons in the penumbra may not depend solely on apoptosis. Fourth, while TH clearly suppresses Xkr8 expression, the upstream regulatory mechanisms remain undefined. Whether TH alters caspase activation, modulates transcriptional programs controlling Xkr8, or affects the stability and trafficking of the Xkr8-Basigin/Neuroplastin complex requires further investigation [55]. In addition, only male C57 mice were used in the present study. Considering the potential influence of sex differences on therapeutic efficacy [56], it remains unclear whether TH exerts similar protective effects on the ischemic penumbra in female mice. Finally, TH has multiple protective effects, other pathways could contribute to protection, including modulation of mitochondrial integrity [57], inflammatory signaling [[58], [59], [60]], or vascular responses [16], which were not systematically examined here.

In the future, more accurate penumbra assessment techniques should be developed to accurately quantify the changes in the size and dynamics of the penumbra volume. The incorporation of high-resolution time-history imaging to follow PS exposure and phagocytosis interactions in vivo would provide key supporting evidence for apoptotic pathways. Direct assays of microglial engulfment, combined with manipulation of specific “eat-me” signals, will be essential to confirm that TH acts through suppression of phagoptosis rather than upstream apoptosis alone. Mechanistic studies dissecting how TH downregulates Xkr8—whether through transcriptional repression, reduced caspase signaling, or impaired scramblase complex assembly—will refine the molecular mechanisms proposed here. In addition, further validation studies with larger cohorts including both male and female mice are required to determine whether TH exerts similar protective effects in female mice. Finally, extending analyses to chronic phases will determine whether early suppression of PS exposure translates into durable neuronal survival and functional recovery.

In summary, our study identifies Xkr8-dependent PS externalization as a key molecular determinant of neuronal apoptosis in the ischemic penumbra and demonstrates that TH confers neuroprotection by suppressing this pathway. Our findings provide a mechanistically defined link between TH and penumbral protection and suggest that targeting the Xkr8-PS axis may represent a rational and translatable strategy for improving outcomes after ischemic stroke.

## Author contributions

Zhanwei Zhu and Di Wu initiated and conceptualized the study. Zhanwei Zhu, Jian Chen, Jiaxin Hu, Jiachen He, Qi Liu, Jiaqi Guo, Yuncong Li, Jingbei Liu, Shichun Min, Shuaili Xu, Xi Chen and Xiaoduo He conducted experiments. Di Wu, Meimei Tao and Jian Chen provided resources. Zhanwei Zhu, Jian Chen and Jiaxin Hu drafted and wrote the paper.

## Declaration of competing interest

The authors declare that they have no competing interests.

## Figures and Tables

**Figure:**
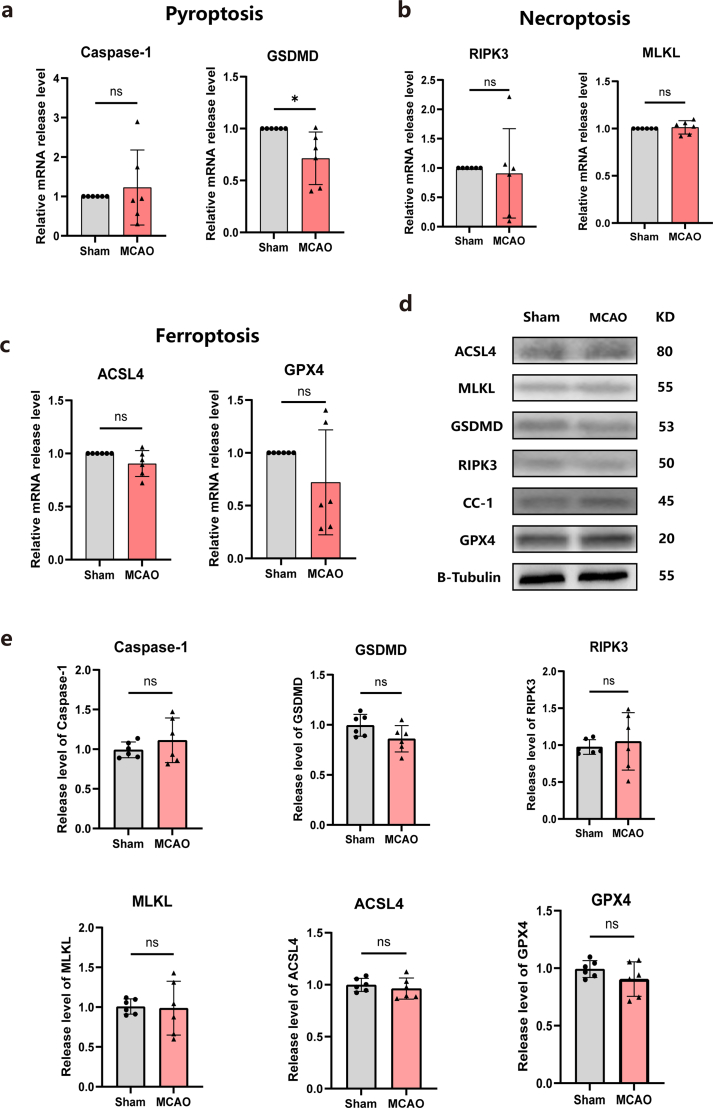
Fig. S1**Validation of the levels of key factors in cell death signaling in the ischemic penumbra**. (a-c) Results of mRNA levels of key factors of pyroptosis, necrosis and ferroptosis in the ischemic penumbra region (n = 6). (d-e) Results of protein levels of key factors of pyroptosis, necrosis and ferroptosis in the ischemic penumbra region (n = 6). ∗*P* < 0.05.

**Figure:**
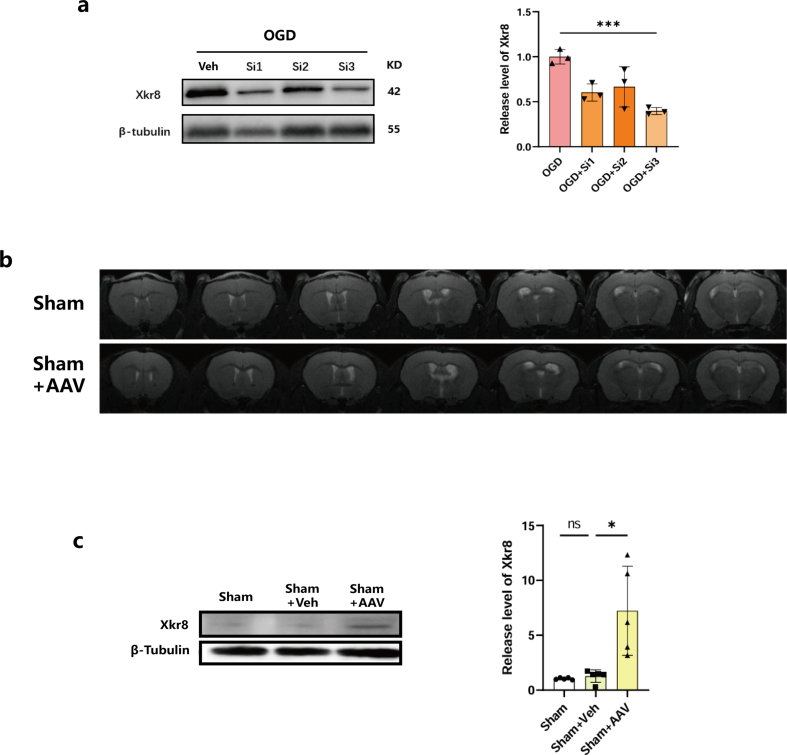
Fig. S2**The effectiveness of Xkr8 knockdown model and Xkr8 overexpression model was verified.** (a) Validation results of Xkr8 knockdown model protein levels in primary neurons (n = 3). (b) The MRI results of AAV injection into the lateral ventricle of mice showed that no brain injury was observed. (c) Results were verified by Xkr8 protein overexpression (n = 5). ∗*P* < 0.05, ∗∗∗*P* < 0.001.
