# Supplementary material for: Therapeutic hypothermia protects the ischemic penumbra via Xkr8
Source: Neurotherapeutics. 2026 Jun 20;23(4):e00948. doi: 10.1016/j.neurot.2026.e00948 (PMC13310642; doi:10.1016/j.neurot.2026.e00948)
Supplement: Multimedia component 1 [file mmc1.docx]

**Table S1 Antibodies.**

| **Antibodies** | **Source** | **Identifier** |
| --- | --- | --- |
| NeuN | Santa Cruz Biotechnology | 92590 |
| MAP-2 | Santa Cruz Biotechnology | sc-20172 |
| ICAM-3 | Santa Cruz Biotechnology | sc-390307 |
| Alexa Fluor™ 555 anti-Rabbit | Thermofisher | A-31572 |
| Alexa Fluor™ 555 anti-Mouse | Thermofisher | A-31570 |
| Alexa Fluor™ 488 anti-Rabbit | Thermofisher | A-21206 |
| Alexa Fluor™ 488 anti-Mouse | Thermofisher | A-21202 |
| Xkr8 | Thermofisher | PA5-98929 |
| β-Tubulin | cell signaling | 2146S |
| TUNEL | YESEN | 40307-B |
| PSIVA | EMD Millipore Corp | 16-256 |
| CRT | Selleckchem | F0472 |
| ANO6 | cloud-clone | PAF813Hu01 |
| TMEM30A | abcam | AB217330 |
